# Advantages of Using Triboscopic Imaging: Case Studies on Carbon Coatings in Non-Lubricated Friction Conditions

**DOI:** 10.3390/ma15124317

**Published:** 2022-06-18

**Authors:** Lars Lorenz, Stefan Makowski, Volker Weihnacht, Matthias Krause, Andrés Fabián Lasagni

**Affiliations:** 1 Institute for Manufacturing Technology, Technische Universität Dresden, George-Bähr-Strasse 3c, 01069 Dresden, Germany; andres_fabian.lasagni@tu-dresden.de; 2 Fraunhofer Institute for Material and Beam Technology IWS, Winterbergstrasse 28, 01277 Dresden, Germany; stefan.makowski@iws.fraunhofer.de (S.M.); volker.weihnacht@iws.fraunhofer.de (V.W.); 3 Institute of Ion Beam Physics and Materials Research, Helmholtz-Zentrum Dresden-Rossendorf, Bautzner Landstrasse 400, 01328 Dresden, Germany; matthias.krause@hzdr.de

**Keywords:** DLC, ta-C, a-C, friction, wear

## Abstract

Triboscopy focuses on the analysis of the temporal evolution of a tribological system, combining local and time-resolved information, most commonly the evolution of friction. In this work, this technique is applied on measurements, which were carried out with a custom-built ultra-high vacuum tribometer in ball-on-disc configuration. Based on these experiments, an extended classification to distinguish different triboscopic features is suggested, depending on the persistence in both track position and time: Uniform, Global, Local, and Sporadic. Further, a filter technique for quantifying triboscopic data regarding this classification is introduced. The new and improved triboscopic techniques are applied to various dry friction measurements of hydrogen-free carbon coatings under varying humidity and pressure. The resulting specific triboscopic features are correlated to wear phenomena, such as counter body coating abrasion, inhomogeneities in the wear track, non-uniform track wear, stick-slip and debris in the contact area, demonstrating the increased analysis and monitoring capabilities when compared to conventional friction curves and wear track images.

## 1. Introduction

Friction analyses of tribological experiments are commonly reduced to friction curves, which represent the coefficient of friction, e.g., by averaging friction coefficients during a certain number of cycles or by picking specific points in a cycle. Wear analyses are usually also performed ex situ after the tribological test, and the temporal evolution of wear must be reconstructed by correlation with the friction curves. A separate examination of different parts of the wear track regarding temporal progression is therefore not possible. To overcome this obstacle, in situ methods with e.g., microscopes, Raman spectrometers, and FTIR spectrometers have been applied, but are either expensive or limited to certain counter body materials [1,2,3]. In addition, these are often not feasible for vacuum tribometer setups.

These analytical shortcomings can be overcome for most tribological tests using triboscopy. The term triboscopy has been formulated by M. Belin nearly 30 years ago, in which the tribological information provided by a tribometer was coupled with the numerical process of the recorded information, creating an image [4,5]. This idea usually manifests in the two-dimensional visualization of, e.g., friction for both position and time during or after a tribological experiment. However, any other kind of information that can be obtained with sufficiently high resolution can be also chosen for triboscopic imaging. In this frame, alternative parameters such as the electrical resistance have been utilized as reported by Wahl et al. [6]. In that work, they demonstrated the region-specific evolution of the contact resistance for a steel surface in contact with MoS_2_, offering insights which cannot be gained from analyzing only the evolution of the friction coefficient. The possibility to attribute changes to specific positions in the wear track has also been demonstrated [7].

Triboscopical methods are also applicable to measurements obtained from non-traditional tribometers, e.g., friction force microscopy (FFM) [8,9,10,11]. Nanofriction experiments have been used to demonstrate the rearrangement of surface atoms in situ [12], study stick-slip behavior at the nanoscale [13], investigating the orientation-dependent friction of organic crystal surfaces [14], and also to measure wear on atomic scales [15]. 

Within different coatings systems, that can be used for improving the tribological performance of parts, hydrogen-free tetrahedral amorphous carbon (ta-C) is a form of carbon (also sometimes called diamond-like carbon (DLC)) with a high degree of sp^3^-hybridized atoms (exceeding 50 at%). Furthermore, the degree of sp^3^-hybridized atoms determines mechanical properties of the material, including density, hardness, and Young’s modulus, making ta-C the hardest type of amorphous carbon [16,17]. Tetrahedral amorphous carbon coatings can be produced by physical vapor deposition techniques that provide high ionization of the carbon. In addition to its outstanding mechanical properties, ta-C coatings also show a beneficial tribological behavior (low friction and wear) that enables its application as friction-reducing and/or wear protection coatings on highly stressed, oil-lubricate components, e.g., on automotive engine components [18,19,20]. On the other hand, under dry friction conditions, ta-C exhibits widely different friction and wear behavior depending mostly on the presence of water vapor [21]. Water vapor leads to the termination of free surface bonds, reducing friction significantly, and accordingly, its absence—e.g., in vacuum—to catastrophic friction and wear [22]. However, there are exceptions to this depending on the counter body material. In the case of a brass counter body to a ta-C coating, it has been shown that low friction and wear can be achieved in vacuum [23].

The use of triboscopy for localized friction analysis has been suggested by dos Santos et al., along with showing several relevant applications [24]. Long et al. used triboscopy in their superlubricity study of ta-C with glycerol to define the valid region within the lateral wear track with sufficiently high speed [25]. Another application in the field of carbon films was demonstrated by Fontaine et al. in their research on the healing effect of hydrogen gas on a-C:H films [26]. However, up to now a comprehensive formal classification of triboscopic features along with a quantitative analysis has not been attempted, and acceleration information has until now not been incorporated.

In this study, we apply the concept of triboscopy to dry friction experiments done on hydrogen-free carbon coatings and extend the methodology with the use of acceleration information. The main objective is to present the methodology of triboscopy on tribological measurements of carbon coatings in a way that can be transferred to future experiments. Therefore, the correlation of triboscopic features with wear and/or friction phenomena is explored. Further, we present a novel method for quantifying the contribution of each triboscopic feature to the overall friction pattern.

## 2. Materials and Methods

### 2.1. Materials

Different hydrogen-free amorphous carbon coatings were deposited using the Laser-Arc technique [27] with a commercial PVD coating system, including a-C, ta-C, boron nitride-doped ta-C (ta-C:B:N) and copper-doped ta-C (a-C:Cu). The a-C and three different ta-C coatings, hereinafter referred to as ta-C_1_, ta-C_2_, and ta-C_3_, were produced from a pure graphite cathode, by using different deposition parameters, such as bias voltage and temperature, resulting in different mechanical properties, as shown in Table 1. The three types of ta-C coatings have very similar properties and are distinguished only for the sake of completeness. In the case of the ta-C:B:N coating, a composite cathode, made of 80% graphite and 20% boron nitride was used, while for the a-C:Cu coating the corresponding cathode contained 90% graphite and 10% copper. The deposition of the doped coatings has been described in [28].

Steel disks made of hardened low-alloy chromium steel (100Cr6, EN 1.3505) with a diameter of 24 mm and a thickness of 6.9 mm were used as substrates for the different coatings. The samples were mounted in a two-fold rotation arrangement corresponding to an industrial coating of tribological components. Prior to carbon coating, an Ar+ ion etching was performed, using a hollow-cathode plasma source. After etching, a chromium interlayer with thickness of about 100 nm was deposited by magnetron sputtering. 

After deposition, different characteristics of the coating systems were determined on steel reference samples. The coating thicknesses were measured by crater-grinding method according to EN ISO 26423:2016. Mechanical properties were derived from nanoindentation experiments, using a ZwickRoell ZHN nanomechanical test system (Ulm, Germany). Measurements were taken with a Berkovich tip, loaded up to 100 mN with quasi continuous stiffness method (QCSM). Both the Young’s modulus and hardness of the coatings were acquired as described in EN ISO 14577-4:2016. However, for extrapolation of coating indentation modulus, a sigmoid fit model was used instead of the proposed linear fit according to [29]. The B, N, and Cu content in the ta-C:B:N and a-C:Cu coatings were obtained by EDS measurement, using JEOL 6610 + X-Max 80 mm^2^ (JEOL, Akishima, Japan and X-MAX 80 from Oxford Instruments plc., Abingdon, United Kingdom). The coating properties are summarized in Table 1. Prior to tribological testing, the deposited coatings were polished with diamond suspension to a roughness of Ra < 20 nm.

For the tribological tests, an array of different counter body materials was used, as listed in Table 2.

### 2.2. Tribological Testing

The tribological measurements in this work were carried out using an ultra-high vacuum tribometer (Tetra Basalt UHVT-14, Ilmenau, Germany) where a fixed ball is pushed against a rotating disk. The measurements were performed using a normal load of 5 N, a velocity of 3 mm⸱s^−1^ with sliding distances between 12 m and 60 m. The angular position and coefficient of friction were sampled at 1 kHz rate, written into files and processed further as discussed later with custom C++ and Python programs. Along with the triboscopic images, two two-dimensional graphs will be shown: one averaged over all positions, corresponding to traditional friction curves and one averaged over all cycles, showing the overall differences between the positions. All the experiments were performed in dry conditions without adding lubricants. Different atmospheres were employed, including dry (1% RH) and humid (90% RH) air, nitrogen (0% RH) and vacuum (4∙10^−7^ mbar, 10^−2^ mbar, 10 mbar) under ambient temperatures. SEM images have been prepared with a JEOL 6610 microscope (JEOL, Akishima, Japan).

### 2.3. Triboscopic Classification

From the recorded raw data, triboscopic images were prepared. Since a rotational setup was used, the position of the ball in the wear track was defined as angular position. In the following, both friction and acceleration triboscopic images will be discussed. In case of the acceleration triboscopy, the experiments had to be run in closed loop control. For the triboscopic images shown in this publication, the absolute acceleration was calculated from the position and time information after the experiment, which introduces slight noise into the triboscopic images. All the data transformation and visualization were done with a custom software due our choice of a very high (1 kHz) temporal resolution.

**Figure 1:**
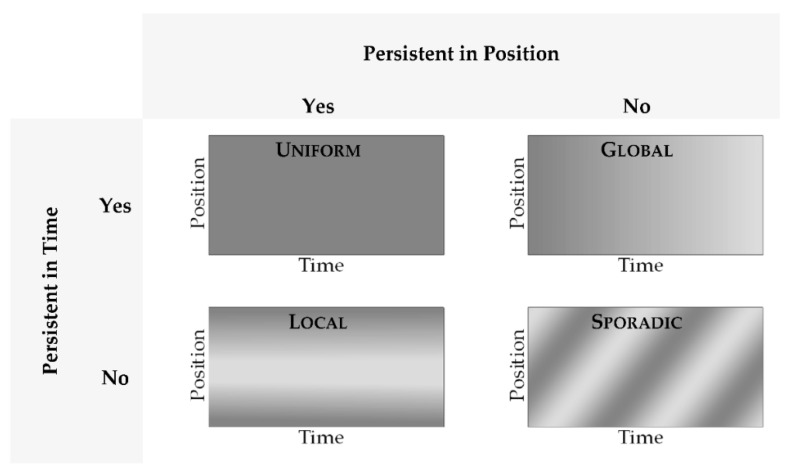
Schematic representation of classification of triboscopic features with an example given for each case. Uniform: No noteworthy features. Global: Features are persistent over the entire track. Local: Features are at specific positions inside the track and persist over many cycles. Sporadic: Features do not persist in either cycle or time.

The triboscopic features were classified as shown in Figure 1. The terminology is partly derived from previous publications, which used the terms “Global” and “Local” [6]. For the Global case, the triboscopic value changes over time, but does so for all positions in the track equally. This hints at a structural change on the moving counter body, e.g., the formation of a tribolayer. For the Local case, there are persistent features at certain positions, which hint changes on the lower sample side.

An extension to the classification, which is proposed here, is based on the combination of the persistence of a certain property (e.g., friction coefficient) in both time and position. For the Uniform case, no significant change can be observed during the experiment. Lastly, the Sporadic case specifies features, which neither persist in the position nor the cycle.

### 2.4. Quantitative Triboscopy

An approachable way to quantify triboscopic features can be achieved by filtering the triboscopic data by direction. This can be performed with two-dimensional Fourier transformation and filtering the frequency bins in the frequency domain by direction.

The following limits are proposed:

- Global: $\left(\pm \frac{\pi }{2}-\frac{\pi }{32}\right)rad$ to $\left(\pm \frac{\pi }{2}+\frac{\pi }{32}\right)rad$
- Local: $\left(\frac{\pi }{2}\pm \frac{\pi }{2}-\frac{\pi }{32}\right)rad$ to $\left(\frac{\pi }{2}\pm \frac{\pi }{2}+\frac{\pi }{32}\right)rad$
- Sporadic: $\left(\pi \pm \frac{\pi }{2}+\frac{\pi }{32}\right)rad$ to $\left(\frac{3\pi }{2}\pm \frac{\pi }{2}-\frac{\pi }{32}\right)rad$
- Uniform: no limits

The choice to use a tolerance of $\pm \frac{\pi }{32}$ has been made empirically to allow for slight changes over several cycles or positions, respectively. In the sporadic case, a wider range has been chosen to accommodate the often-observed even wider spread of the feature directions compared to global and local. To obtain the filtered triboscopic images shown in this publication the following transformation steps have been used:

1. Subtracting mean value
2. Discrete Fourier transform
3. Filtering amplitudes by direction
4. Inverse Discrete Fourier transform

**Figure 2:**
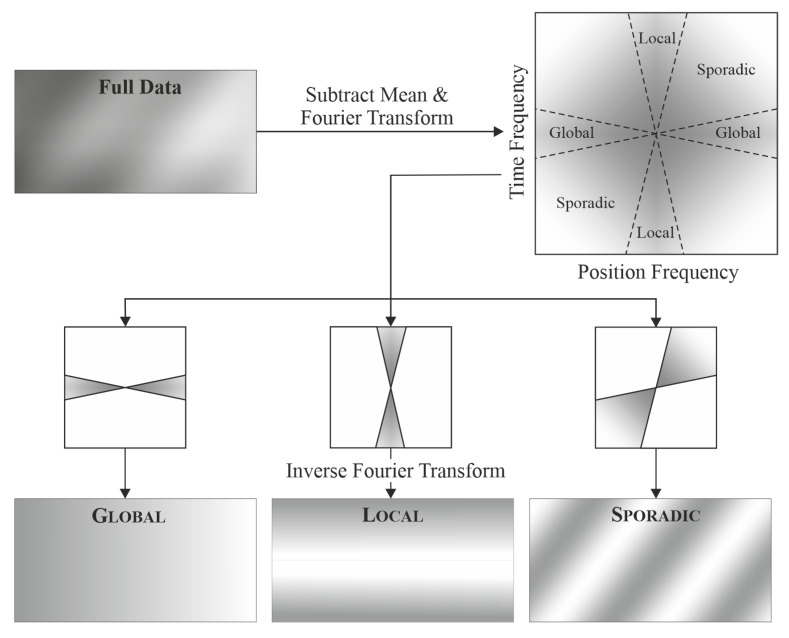
Steps for obtaining directionally filtered triboscopic images from the triboscopic data.

The schematic procedure is illustrated in Figure 2.

From the filtered triboscopic data it is possible to gain a comparable and characteristic value indicating the magnitude in the specific direction by taking the mean of all points in the filtered data for both position and time. These values are called directional mean coefficient of friction and denoted as such: $\bar{COF_{global}}$, $\bar{COF_{local}}$, and $\bar{COF_{sporadic}}$ respectively.

## 3. Results and Discussion

### 3.1. Case Studies

Triboscopy will add valuable information to tribological testing when its features can be correlated with specific friction and wear phenomena. In the following case studies, such a correlation is presented and discussed, using selected triboscopic data obtained from dry friction experiments on various carbon coatings.

Overall, it is demonstrated that advanced triboscopy enables the ability to verify or increase information from the experiment.

#### 3.1.1. Abrasion of Ball Coating

The global case can assist with identifying the exact moment a coating is fully worn and the substrate exposed on the moving part. This scenario is typical for studying thin coatings that do not endure the testing time or also might delaminate.

**Figure 3:**
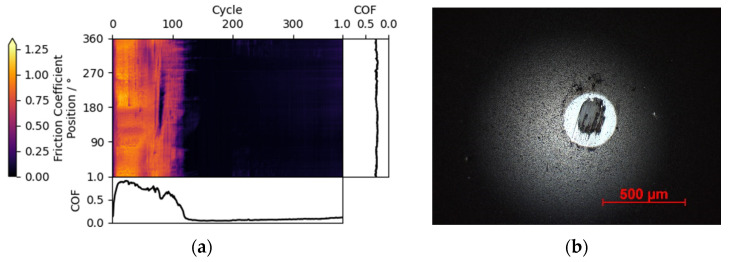
(**a**) Friction triboscopic image for an a-C coating against a ta-C_1_ coated ball in 0.01 mbar vacuum, exhibiting global features. (**b**) Micrograph of ta-C_1_ counter body with worn-through coating and protective tribolayer (black inside white).

In the presented case, a tribo-measurement of a ta-C_1_ coated ball against an a-C coating at 0.01 mbar vacuum is examined with the triboscopic image given in Figure 3a. A drop in friction starting around cycle #100 can be observed in the friction curve. In the triboscopic image, this change is confirmed to be Global, meaning that the change in friction is likely due to a change occurring on the counter body.

The optical microscope micrograph in Figure 3b confirms that the coating is indeed worn through and that a protective tribolayer has formed over the exposed steel area. This leads to the conclusion that the initial ta-C/a-C contact is characterized by high friction and thus wear, which decreases significantly as soon as the tribolayer forms, leading to a Global feature.

Consequently, in this case, the a-C coating on the disk is softer than the ta-C on the ball. Thus, coating failure by wear would have been feasible for the disk coating as well, but most likely would have shown up as Local feature upon initiation. Instead, a strong assumption on the wear could be made during the course of the experiment.

#### 3.1.2. Inhomogeneities

**Figure 4:**
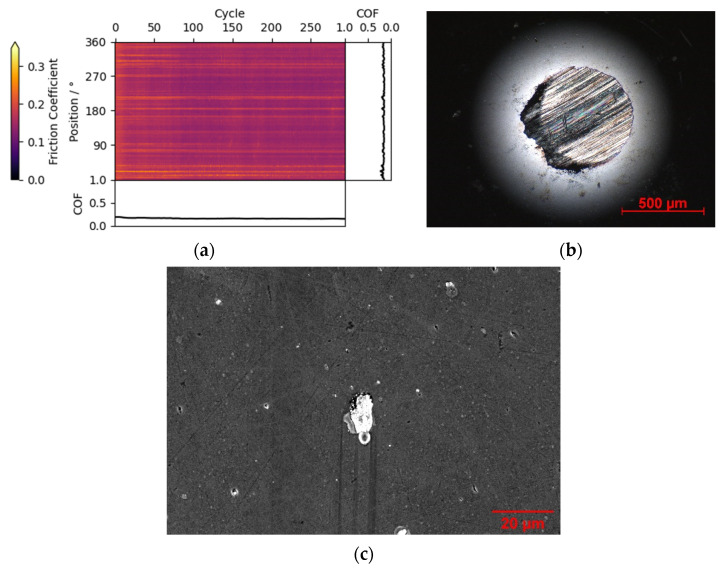
(**a**) Friction triboscopic image of ta-C:B:N vs. a steel ball counter body in humid air with pronounced local features. (**b**) Micrograph of worn counter body. (**c**) SEM image of wear track with visible transferred steel particle.

The local case can assist in attributing the triboscopic measurand to a specific position in the wear track. An example is given in Figure 4a, with a triboscopic image from a non-lubricated friction experiment from a ta-C:B:N coating against steel ball in humid air. In humid conditions, a stable coefficient of friction below 0.1 and no measurable wear are expected for pure ta-C coatings [22]. Instead, a coefficient of friction of 0.15 and high counter body wear (Figure 4b) are observed. With the triboscopic image most of this increase of friction can be attributed to certain local regions inside the track (Figure 4c), which are not visible in the conventional friction curve alone. The cause of these regions has been attributed to transferred steel counter body debris at holes in the surface, which might have resulted from arcing damage.

#### 3.1.3. Non-Uniform Track Wear

**Figure 5:**
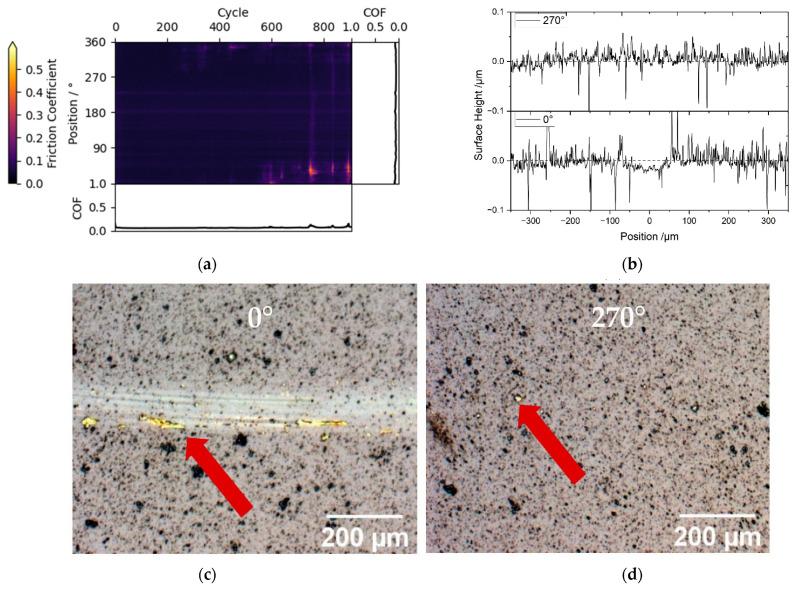
(**a**) Friction triboscopic image of a ta-C_2_ disk vs. a brass counter body in humid air with local features; (**b**) shows the track profile for both 270° and 0°, with 270° showing slight wear and 0° showing no wear; (**c**,**d**) wear track micrographs of experiments: (**c**) 0° position; (**d**) 270° position, with exemplary spots of brass highlighted with red arrows. The transferred brass is clearly visible in the first wear track, where the track is measurably worn, indicating a correlation between brass transfer and subsequent carbon coating wear.

Another use case for local triboscopy is the in situ detection of non-uniform wear track behavior, which can evolve during the experiment. An example is given in Figure 5a for a brass ball sliding against ta-C_2_ coated disk in humid air. After approximately 600 cycles, the friction starts to spike at the position around 45°. This increase in friction is correlated with the localized transfer of counter body material to the ta-C surface, leading in turn to sliding of brass against brass in these positions. This brass transfer is clearly visible in the wear tracks in Figure 5c, and large brass fragments, which have already transferred, can be seen in the 270° micrograph in Figure 5d. Stylus measurements of the two positions, shown in Figure 5b, confirm that the transfer precedes the friction increase, since the track at 270° is not yet worn despite showing the aforementioned brass transfer.

#### 3.1.4. Detection of Stick-Slip

**Figure 6:**
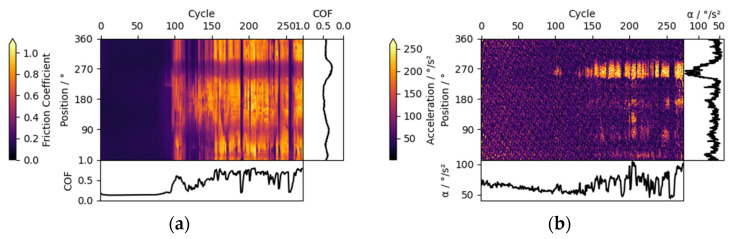
Triboscopic images for a ta-C_3_ coating vs. a Cu counter body in 4∙10^−7^ mbar vacuum, showing regions of stick-slip at position 270°, starting at cycle 100; (**a**) friction triboscopic image; (**b**) acceleration triboscopic image.

Stick-slip can be detected from the combination of friction and acceleration triboscopy, manifesting itself as regions of horizontal “zebra”-patterned friction features and as regions of high acceleration. Such behavior is shown in Figure 6 around position 270°, starting at cycle 100. The pattern emerges due to the alternating sticking (high deceleration and high tangential force) and slipping (high acceleration and low tangential force) which, considered together, are conclusive evidence for stick-slip.

In the presented experiment, a Cu ball was slid against the ta-C_3_ coating at 4∙10^−7^ mbar vacuum. While the sudden increase in friction can be seen for the whole wear track, high acceleration is found only for a local and steady position of the wear track. It is concluded that the first and local occurrence of stick-slip destroyed the passivated low friction surface on the Cu ball, leading to high friction over the whole track. Later during the experiment, friction frequently and sharply dropped to low levels for short time, e.g., around cycle 190 and 260, likely due to the removal of the transferred brass. However, such conditions were not stable and stick-slip occurred again at same disk positions.

It must be noted that pronounced stick-slip in our case is exacerbated by low stiffness of the measurement setup.

#### 3.1.5. Debris in Contact Area

**Figure 7:**
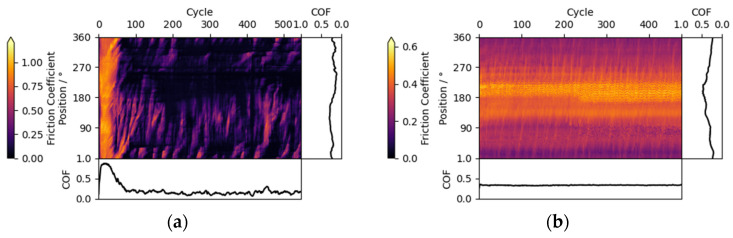
(**a**) Friction triboscopic image demonstrating sporadic features from wear debris for a ta-C_3_ coating vs. a ta-C_1_ counter body in a 10 mbar vacuum; (**b**) friction triboscopic image for a steel disk vs. a steel counter body with added sepiolite wear particles. The local feature at ca. 180° to 225° is caused by the increased wear caused from the particles dropped in this area.

As detailed previously, the Sporadic case hints at changes which are neither depending only on the sample nor the counter body. One example with pronounced Sporadic features is given in Figure 7a, where diagonal features appear in the direction of movement. These features can be attributed to wear particles, which are carried a short distance in the contact and picked up again in the next cycle. In conventional analysis of the friction curve such behavior is usually not visible.

In the presented experiment, two superhard ta-C coatings were tested at 10 mbar vacuum, ruling out adhesion or plastic coating deformation in the contact. Instead, wear is likely to occur from brittle failure of the coating and detachment of embedded growth defects, leading to loose and hard particles in the contact area.

To confirm the origin of such features from wear debris, further experiments with artificial wear particles were performed. Sepiolite particles were chosen as a non-metal particle with moderate hardness and introduced into a contact of a steel ball sliding against a steel disk in ambient air. The resulting triboscopic image is shown in Figure 7b, exhibiting the same diagonal sporadic features and confirming the link between wear debris and sporadic features.

**Figure 8:**
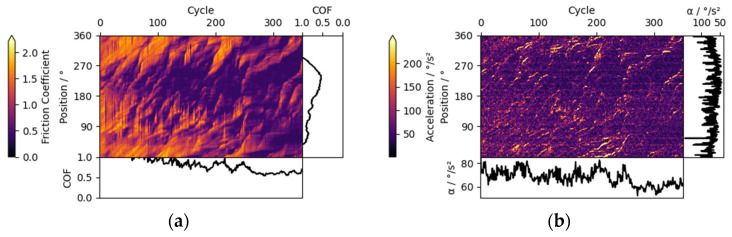
Triboscopic image for a ta-C_3_ coating vs. a ta-C_1_ counter body in a nitrogen atmosphere. (**a**) Friction triboscopic image. (**b**) Acceleration triboscopic image.

Additionally, also acceleration triboscopy can be taken into consideration for the detection of wear debris, which is shown in Figure 8b. The well-defined sporadic high acceleration features are clearly visible and match up quite well with the edges of the friction image. In contrast to the previous stick-slip features, these particle-induced features are thinner, because they only occur when wear debris is picked up by the contact.

In the triboscopic image given in Figure 8a, there is another interesting phenomenon to be found, showing that friction spikes continue for part of the track (vertical lines, fading out towards the top), but also carry over to the next cycle. This detail could be interpreted in such a way that a part of the material is carried with the ball, while the rest is remaining on the lower sample surface. Furthermore, the Sporadic features widen during transport, eventually disappearing, suggesting that they continuously split up in smaller pieces until they do not affect COF anymore or are transported out of the contact.

In addition, it can be speculated that the speed of the material transport and the width of the features on the triboscopic map compared with the actual contact diameter measured on the ball can hold further information on the wear process.

For example, an ideal round and brittle wear particle is likely to roll in the contact between disk and ball. From entrapment until release of the contact, the particle will interact for the double distance of the contact diameter found on the counter-body ball and then influence the friction signal. During interaction, the particle will travel the single distance of the ball contact diameter.

Opposed to that, wear debris with plastic behavior will most likely stick to either surface or just a small amount of it will travel through shearing. Therefore, both interaction distance and speed of transport are lower.

While ideal cases will not be observed in real experiments, the speed of transport of Sporadic features might hint at the brittle or plastic nature of the wear particles.

### 3.2. Quantitative Triboscopy

**Figure 9:**
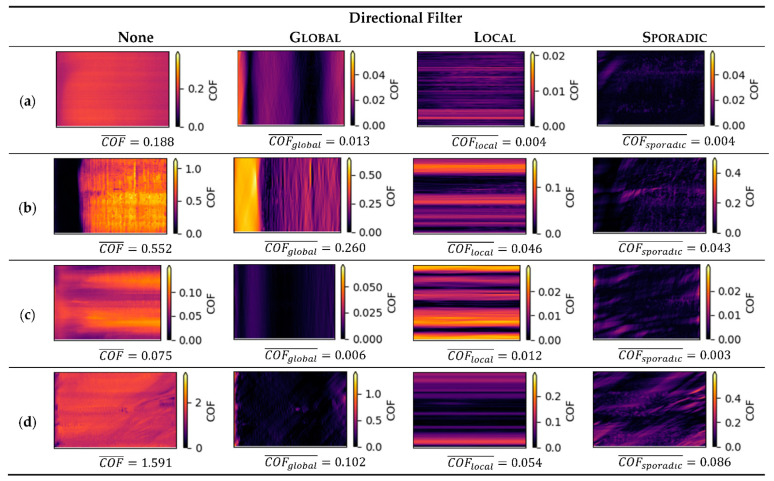
Triboscopy images for the proposed directional filtering technique Global, Local, and Sporadic. Below every filtered image, the directional mean coefficient of friction is given, with column “None” being the original images, including the mean coefficient of friction, which also corresponds to the Uniform case. Scales for friction coefficient are individually scaled for each image to emphasize the quality of friction features of each contribution, reducing the visibility of their quantitative contribution. Tribological testing cases given as examples in rows are (**a**) a-C:Cu, steel ball, humid air, with no dominant directional features; (**b**) ta-C_3_ coating, steel ball, ambient air with strong Global and Sporadic features; (**c**) ta-C_3_ coating, ta-C_1_ ball, ambient air, with mostly local features; (**d**) ta-C_3_ coating, ta-C_1_ ball, nitrogen, with sporadic features.

To gain a more complete impression on how different features appear under the different filters, several examples of friction triboscopic images are given in Figure 9 with their respective filtered features and friction coefficients. None of the studied examples had pure forms of directional features, but contained each of them. However, their contribution to the overall friction varies significantly.

It becomes clear that Uniform features dominate the friction process by quantity, Furthermore, it should be noted that the overall Uniform friction level strongly depends on the surface materials and experimental conditions, so it can vary from experiment with rather low friction (as in Figure 9c) to extremely high friction (as in Figure 9d). Thus, directional features such as Global, Local, and Sporadic must always be discussed in relation to the Uniform feature.

Most dominant directional feature in our studied examples were Global (with $\bar{COF_{global}}/\bar{COF}=0.47\;$for Figure 9b), followed by Local (with $\bar{COF_{local}}/\bar{COF}=0.16\;$for Figure 9c) and Sporadic (with $\bar{COF_{sporadic}}/\bar{COF}=0.08\;$for Figure 9d).

Whereas in this overview all examples stem from completely different experiments investigating different tribological problems, application of such quantification within the same series of measurements allows to precisely differentiate and quantify tribological features and their quantitative contributions.

## 4. Conclusions

In this work, the triboscopy method was evaluated and extended with regard to classification and quantification. For demonstration of the benefits, it was applied to various non-lubricated friction experiments with different amorphous carbon coatings, counter body materials and atmospheres, thus obtaining a broad spectrum of different friction and wear behavior.

For interpretation of the measured data, an extended classification was proposed based on previous publications, which assigned features from triboscopic images into four distinct groups Uniform, Global, Local, and Sporadic, depending on the feature direction in position and time.

Based on this classification, a directional filtering technique for obtaining quantitative triboscopic values was established. For the first time, acceleration triboscopy is studied in addition to friction triboscopy. In contrast to other measurands, such as contact resistance, acceleration is easy to obtain with conventional tribometer setups from the drive system.

Using the presented methods, several case studies are discussed by correlating wear and friction processes with apparent triboscopic features. Processes such as abrasion and coating failure on the ball, detection of coating inhomogeneities, non-uniform track wear, stick-slip behavior, and detection and behavior of debris in the contact were studied and discussed. It was shown that many friction and wear processes can be studied in detail, allowing direct correlation of tribological and triboscopy features in both location and time, far exceeding the possibilities from conventional friction progression curves.

While in the context of this publication the correlations were made after the experiments, the use of these findings for monitoring in in situ experiments is strongly suggested.

In addition to direct correlation, quantification of triboscopic features is possible by application of defined filtering methods, to study the influence of different features with regard to the overall friction coefficient.

Given the fact that many tribometers are capable of providing high-rate data for friction and position, real-time triboscopic evaluation of such data can give detailed in situ access to tribological processes without adding any costly instrumentation.

In this work, it is shown that advanced triboscopic methods can enhance information collected from tribological experiments to study location, chronology, and quantity of special tribological events, such as stick-slip and behavior of wear particles.

## Figures and Tables

**Table 1:** Overview of tested amorphous carbon coatings, including thickness, hardness, Young’s modulus and composition.

Coating	Thickness /µm	Hardness /GPa	Young’s Modulus /GPa	Composition /at%
a-C	3.2	23	238	C: 100
ta-C_1_	3.7	40	456	C: 100
ta-C_2_	4.0	51	519	C: 100
ta-C_3_	4.3	49	587	C: 100
ta-C:B:N	1.4	35	400	C: 85.7 B: 13.1 N: 0.8 Other: 0.6
a-C:Cu	1.4	23	271	C: 84.9 Cu: 14.3 Other: 0.8

**Table 2:** Overview of tested balls used as counter bodies with their respective material composition and ball diameter.

Name	Material	Ball Diameter
Steel	100Cr6	10 mm
Copper	Cu	10 mm
Brass	CuZn35	10 mm
Bronze	CuSn6	9.525 mm
ta-C_1_	see Table 1	10 mm

## Data Availability

The data presented in this study are openly available in Zenodo at https://doi.org/10.5281/zenodo.6657089.
